# Judging Offenders With Intellectual Disabilities: Systematic Review of Criminal Justice System Professionals' Expressed Views and Attitudes Towards Offenders With Intellectual Disabilities

**DOI:** 10.1111/jir.13252

**Published:** 2025-05-19

**Authors:** Georgia Powell, Kate Blake‐Holmes, Adela Petrache, Rebecca Turrell, Peter Beazley

**Affiliations:** ^1^ Department of Clinical Psychology, Norwich Medical School University of East Anglia Norwich UK; ^2^ School of Social Work University of East Anglia Norwich UK; ^3^ Berkshire Healthcare NHS Foundation Trust Berkshire UK

**Keywords:** criminal justice system, forensic science, intellectual disability, learning disability, offenders, systematic review

## Abstract

**Background:**

The diagnosis of an intellectual disability is suggested to have particularly stigmatising connotations, particularly within the criminal justice system (CJS). This paper aims to synthesise qualitative studies investigating the attitudes of CJS professionals to people with intellectual disabilities (PWID), specifically offenders with intellectual disabilities, and to appraise their methodological quality.

**Methods:**

A systematic search was conducted using PsychINFO, Web of Science, MEDLINE, EMBASE, CINAHL Complete and EThOS databases. Articles were screened for inclusion by title, abstract and full text to ensure predefined inclusion criteria were met. Individual study quality was rated using the 10‐item Critical Appraisal Skills Programme (CASP) checklist, with the addition of an eleventh item to capture included studies' theoretical underpinnings and optimise the value of the quality appraisal. Thematic synthesis was then used to explore and synthesise the findings of the included studies.

**Results:**

Ten papers were included in the review, spanning 766 participants. Studies included utilised mixed methods surveys (*n* = 3), qualitative surveys (*n* = 1), semistructured interviews (*n* = 3), semistructured focus groups (*n* = 1), unstructured interviews (*n* = 1) and secondary analysis of previously collected research data (*n* = 1). Methodological quality was broadly of a high standard; however, all included papers failed to reflect on the relationship between the researchers and participants. Five themes were identified: conflating diagnoses, perceptions of PWID as offenders, procedural issues affecting PWID, development and maintenance of perceptions, and impact of training.

**Conclusions:**

This review highlights pervasive negative perceptions of offenders with intellectual disabilities within CJS staff groups. Clinician‐ and system‐level factors are considered in the development and maintenance of such attitudes and suggestions made for improving CJS staff perceptions and knowledge of offenders with intellectual disabilities.

## 1 Background

### 1.1 People With Intellectual Disabilities and the Criminal Justice System

People with intellectual disabilities (PWID) may be more susceptible to coming into contact with the Criminal Justice System (CJS) due to communication difficulties, difficulties in emotional regulation and diminished cognitive functioning, which can result in a lack of capacity to understand criminal law and the consequences of one's actions (Chadwick and Wesson 2020; Gendle and Woodhams 2005; Gulati et al. 2021; Hellenbach 2011; Richards and Ellem 2018). The CJS is comprised of multiple agencies that aim to detect and prevent crime, prosecute those accused of committing crimes and facilitate the punishment and rehabilitation of offenders (Criminal Justice Alliance 2024). The prevalence of PWID in police custody in the United Kingdom ranges from 0.5% to 9% of detainees (Bradley 2009) compared with a general population community prevalence of 2.2% of adults (MENCAP 2024a). This is not an isolated issue, as PWID are overrepresented in CJS internationally, evidenced by the demographic composition of prison populations (Fazel et al. 2008; Gulati et al. 2018; Hellenbach et al. 2016) and the profiles of individuals coming into contact with the CJS as suspected offenders (Gulati, Cusack, et al. 2020; Young et al. 2013).

However, despite their disproportionate representation in the CJS, there are issues with underidentification of PWID, as this relies upon adequate information gathering at first point of contact with the CJS and the availability of appropriate assessment through liaison and diversion services (Chester 2018). This is of particular concern for those individuals who have ‘borderline’ intellectual disabilities (ID) or subvert society's stereotypical expectations of PWID. Studies such as Day (1988), Holland et al. (2002), Reed et al. (2004) and Lindsay (2011) have found that defendants with ID are typically characterised as young men with behavioural problems who have endured significant psychosocial disadvantages from early childhood. However, the majority of defendants without ID also present with these characteristics (Holland et al. 2002; Vinkers et al. 2010); therefore, diagnostic conclusions cannot be unduly influenced by these characteristics. Failing to identify PWID when they come into contact with services has serious consequences, such as support needs being unmet before, during and after contact with the CJS (Howard et al. 2015; Murphy et al. 2017); insufficient support and inappropriate questioning in court (Kebbell et al. 2001); and ultimately an increased likelihood of incarceration (Howard et al. 2015). Although they are not homogenous in their support needs, the broad characteristics of PWID include significant communication difficulties, impaired cognitive functioning and impaired adaptive functioning (MENCAP 2024b). The detection of people with support needs in general is quite poor, with many people with psychiatric and/or learning needs never having adequate assessment and treatment (Department of Health and Social Care 2014; Moitra et al. 2022; National Institute for Health and Care Excellence 2023).

Systemic and organisational barriers continue to exist, which results in inequitable treatment of PWID in the CJS with insufficient action taken to support them and protect their human rights (Bradley 2009; Hyun et al. 2013; Lindsay et al. 2011). These are not contemporary concepts; academics have long discussed the disproportionate representation of PWID in the CJS and the relative disadvantages they face (Clare and Gudjonsson 1991, 1993; Gudjonsson et al. 1992; Murphy and Clare 1998). One could argue that this is reflective of the desperate and relatively unchanging socioeconomic inequalities and stigmas faced by PWID in wider society in both the contemporary and historical contexts (Craft 1984; Oshima et al. 2010; Woodward 1955). In the United States, for example, people with disabilities, whether developmental, intellectual or psychiatric, account for approximately one third of deaths in fatal interactions with law enforcement (Perry and Carter‐Long 2016). Despite legislation such as the Police and Criminal Evidence Act (PACE; 1984) in the United Kingdom creating a much‐improved framework for police interviewing, which has been adopted internationally (Schollum 2017), there continues to be a lack of clear pathways, insufficient information sharing and inadequacy of training for CJS professionals for working effectively with PWID (Hayes et al. 2007; Henshaw and Thomas 2011), resulting in PWID being ill‐served in the CJS (Young et al. 2013). It would be remiss not to also consider the emotional and psychological impact of these organisational and systemic failures on PWID who come into contact with the CJS, who often describe feeling frightened, confused and isolated (Gulati, Kelly, et al. 2020; Hyun et al. 2013). Societally, PWID are subject to increased levels of victimisation and social exclusion. In England and Wales, over 79 000 hate crimes were reported against people with disabilities (not exclusively ID) between 2010 and 2023, yet only 4% resulted in a charge or summons (Home Office 2023).

It is important to consider how societal expectations and stereotypes create and maintain views and attitudes towards PWID given the paucity of research specifically focusing on the perceptions of PWID by CJS professionals. Research into general stereotypes in the CJS has some existing foundations for us to draw upon. For instance, it has been found that people largely hold negative biases towards individuals with facial differences (such as disabilities), which are perceived as differing from socially acceptable ‘attractiveness’ (Cash et al. 1977; Efran 1974; Jamrozik et al. 2019; Johnson and King 2017; Solomon and Schoplerl 1978). Additionally, there is the issue of stereotype congruence. As stated by Greenspan (2011, 220), ‘Stereotypes held by judges, juries, and (some) experts are typically grounded in an implicit behavioural and physical phenotype, which is more appropriate to moderate or severe Intellectual Disability, where behavioural and physical characteristics are obvious, and limitations are fairly global’. Put simply, stereotypes pertaining to PWID are likely a significant factor in the underidentification of PWID in the CJS as alleged offenders who do not fit the ID physical stereotype are unlikely to be identified or put forward for assessment and appropriate support.

Even where individuals have received assessment and diagnosis, confusion as to the differentiation of ID, developmental disabilities such as autism spectrum disorder and psychiatric diagnoses is pervasive in the CJS, which goes some way to explaining the failure to identify and adequately support PWID (Bradley 2009; Modell and Mak 2008). How CJS professionals perceive and understand PWID plays a significant role in how suspected offenders experience the CJS and how these cases progress. Research by McAfee et al. (2001) suggests that police officers' perceptions of crime and their responses are influenced by the presence of ID, with officers responding differently to crimes where the victim, alleged offender or both had ID. However, the research did not identify specific patterns of responses, so it cannot be deduced whether police officers' differing responses when faced with PWID are positive, effective or helpful. One might hope that CJS professionals working directly with PWID will hold more positive views compared to the general public, given that attitudes are crucial in how CJS professionals make decisions regarding their behaviour in relation to PWID (Fitzsimmons and Barr 1997; Rosser 1990) and favourable attitudes towards PWID are ‘essential to meeting the police code of ethics which stresses impartiality and respect for human dignity’ (Bailey et al. 2001, 344). Training for CJS professionals can positively impact upon perceptions and understanding of PWID (Bailey et al. 2001; Gardner et al. 2018; Henshaw and Thomas 2011). However, different organisations, jurisdictions and legal systems each have their own approach to mandatory training, and it therefore cannot be assured that any given CJS professional has adequate, or even basic, knowledge and understanding of working with PWID.

### 1.2 Terminology

In this review, the term ‘Intellectual Disability’ (or its abbreviation ‘ID’) is used to describe impaired intellectual abilities and adaptive functioning skills that significantly impact upon an individual's day to day functioning and had an onset prior to adulthood (MENCAP 2024b). Terminology and definitions relating to ID are varied between countries, organisations and professions. Terms such as ‘learning disability’, ‘mental handicap’, ‘mental retardation’, ‘learning difficulties’ and ‘cognitive deficiencies’ are used interchangeably and as both formal diagnoses and informal labels for PWID (Gulati, Kelly, et al. 2020). This issue becomes further complicated when comparing research internationally; for example, what one would refer to in the United Kingdom as a ‘specific learning difficulty’ such as dyslexia is commonly referred to as a learning disability in the United States of America. This in conjunction with disagreement as to the ‘threshold’ of an ID diagnosis when utilising measures of intellectual functioning serves only to further complicate attempts to consolidate understanding on the global scale. For the purposes of clarity and consistency, this review will use the terms ‘intellectual disability’ and ‘people with intellectual disabilities’, or their respective abbreviations (‘PWID’ and ‘ID’), throughout the synthesis. This does not include quotations from studies in which different terminology is used.

### 1.3 The Current Review

This qualitative systematic review offers an exploration into CJS professionals' perceptions of PWID, specifically those who come into contact with the CJS as offenders. Previous systematic reviews have focused on the experiences of PWID in interactions with law enforcement (Gulati, Cusack, et al. 2020), limited the scope to frontline professionals involved only in the pretrial stages of the CJS (Gulati, Kelly, et al. 2020) or focused more broadly on CJS professional's experiences and associated challenges of working with PWID (Gulati, Kelly, et al. 2020). The aim of this qualitative systematic review was to consolidate the evidence base of the perceptions of CJS professionals of PWID. By understanding existing perceptions, how they are developed and maintained and what may help create a positive shift in perceptions, short fallings within the CJS can be addressed and the experiences of PWID in the CJS can be improved. Given the paucity of research in this area, this review encompassed studies of both child and adult offenders with ID.

## 2 Method

**FIGURE 1:**
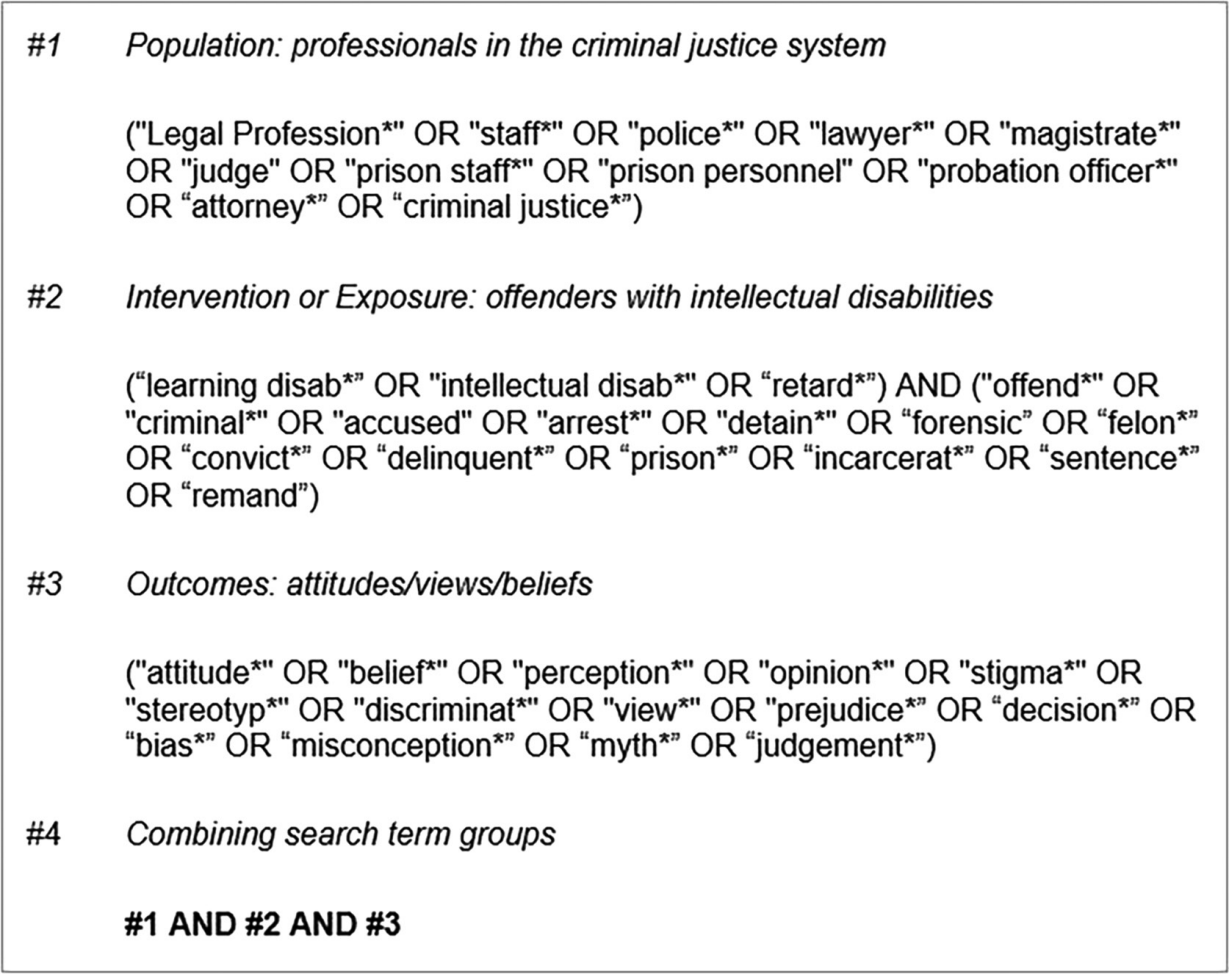
Search terms.

The review question and search terms were developed using the PICO model (Figure 1) and refined following scoping searches of the literature. The review was registered on PROSPERO prospectively (CRD42024506706).

### 2.1 Search Strategy

Six electronic databases (PsychINFO, Web of Science, MEDLINE, EMBASE, CINAHL Complete and EThOS) were searched in February 2024 and updated in December 2024 by one researcher (GP). A predefined search strategy was developed to optimise retrieval of relevant article through incorporation of Boolean operators and truncation.

### 2.2 Selection Criteria

The search aimed to identify qualitative primary research focused upon the attitudes and beliefs of CJS staff groups towards offenders with a diagnosed or suspected ID. This included nonhealthcare professional staff groups who may commonly come into contact with these individuals such as judges, magistrates, parole officers, prison officers, police officers, lawyers and solicitors. The search incorporated all forms of qualitative research methodology, including mixed methods designs. The search included English‐language theses and peer‐reviewed journal articles only. The following exclusion criteria were applied: quantitative studies; studies carried out in setting outside of the CJS (e.g., accident and emergency departments, residential care homes and day centres), studies where data on attitudes or beliefs were not collected; studies that did not focus upon attitudes specifically relating to PWID; studies including nonstaff members within the criminal justice system (e.g., jurors) or professionals not in direct contact with offenders (e.g., housekeeping staff); studies focused solely on healthcare professionals within the CJS; and studies from legal systems not based on Common Law.

Where there was a lack of specificity concerning ID (e.g., studies referencing attitudes to ‘speech, language and communication needs’ or more broadly ‘additional needs’), studies were included if deemed directly relevant to ID following full‐text scrutiny. Limits were set to include articles published between January 1994 and January 2024. As stigmatising attitudes are hypothesised to change over time (Schomerus and Angermeyer 2016), this range was set to explore clinician attitudes within contemporary practice (i.e., over the last 30 years). In order to try to draw in results from across different jurisdictions, a wide scope of terms pertaining to ID was used in the search strategy. This did not, however, include specific learning difficulties such as dyslexia, as this does not align with the aims of the review.

### 2.3 Data Extraction

**FIGURE 2:**
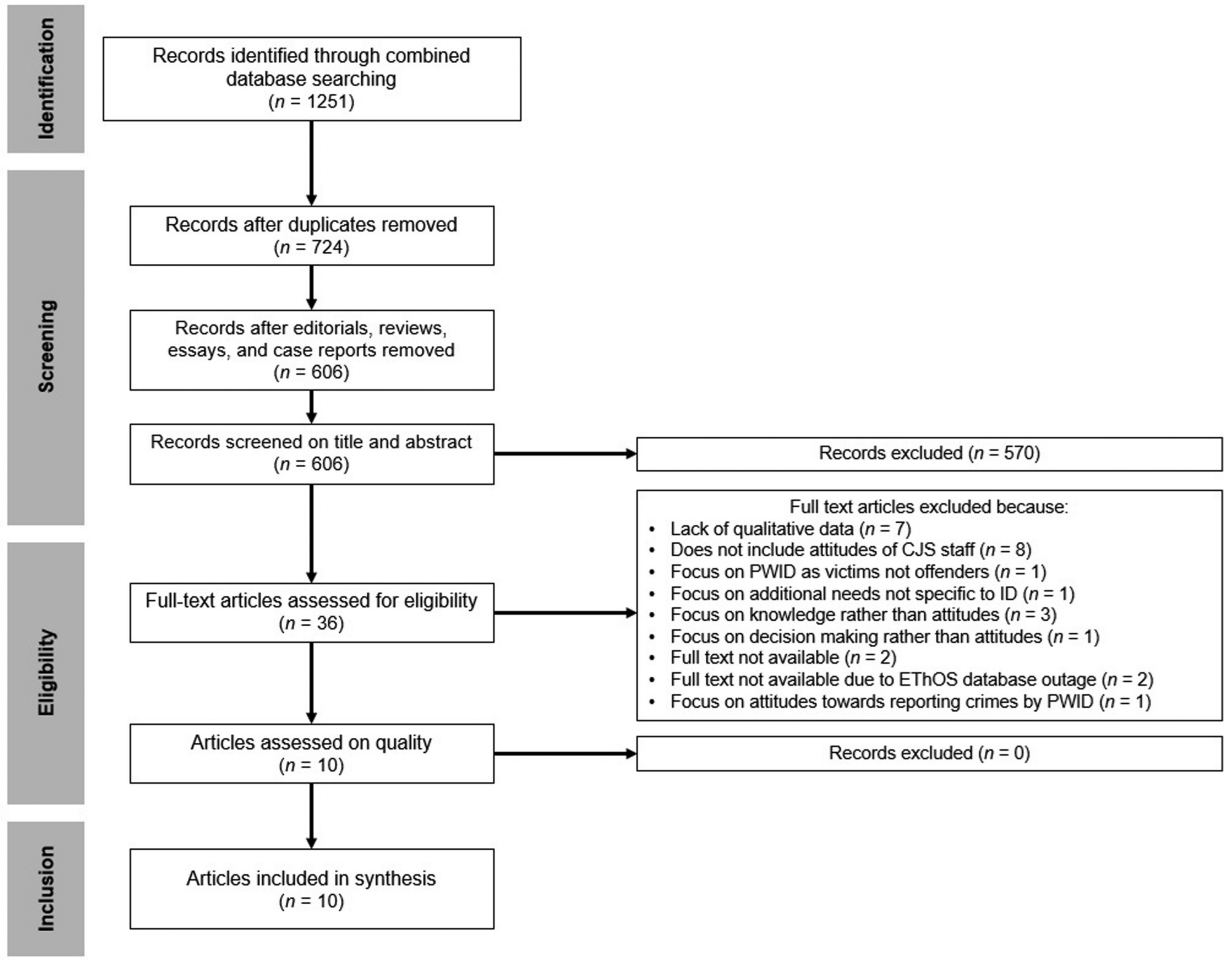
PRISMA flowchart.

Searches were conducted using the above criteria which yielded 1251 results (see PRISMA flowchart depicted in Figure 2). Titles and abstracts were screened by the primary reviewer (GP), with both the primary reviewer and two secondary reviewers (AT and RT) scrutinising 10% of studies at the abstract stage. The primary reviewer assessed all articles at the full‐text screening stage with secondary reviewers assessing 64% of articles at this stage. Discrepancies in reviewer decisions were identified and discussed before progressing to the subsequent stage of screening by referring to the PROSPERO protocol. Records of reviewer decisions and studies included and excluded at each stage were kept using HubMeta (HubMeta 2020). Ten articles fulfilled inclusion criteria and were quality assessed before being included in the data synthesis.

### 2.4 Quality Assessment

All included papers were independently quality assessed by the primary reviewer and a secondary reviewer. Methodological quality of included studies was determined using the CASP checklist and, in instances where there was reviewer doubt or disagreement, cross‐referenced with the five criteria outlined in Stenfors et al. (2020): credibility, dependability, confirmability, transferability and reflexivity. The use of the initial four criteria is well established in qualitative research (Guba et al. 1994); the additional criterion ‘reflexivity’ was incorporated by Stenfors et al. (2020) in order to capture the central role that the researcher embodies in qualitative research, with good quality research exploring, or, at the very least naming, the role of the researcher in the context of the research (Barrett et al. 2020).

When assessing papers using the CASP checklist, a standard of inclusion/exclusion based on scoring was not applied due to the limited research available in this area; therefore, papers of lower quality were not excluded. Regarding CASP Item 10, the reviewers deemed all studies to be valuable given the extremely limited existing knowledge base; therefore, a generic positive response (‘Y’) has been given. An additional eleventh criterion was added to the quality appraisal, as suggested in Long et al. (2020), to optimise the value of the quality appraisal. The quality assessment ratings for the included studies are illustrated in Table 1.

**TABLE 1:** Quality appraisal checklist.

Quality Assessment Criteria	Modell and Mak(2008)	Chadwick and Wesson (2020)	Gulati et al. (2021)	Diamond and Hogue (2022)	Hellenbach (2011)	Henshaw and Thomas (2011)	Eadens et al. (2016)	Gendle and Woodhams (2005)	Cant and Standen (2007)	Richards and Ellem (2018)
1. Was there a clear statement of the aims of the research?	Y	Y	Y	Y	Y	Y	Y	Y	Y	Y
2. Is a qualitative methodology appropriate?	Y	Y	Y	Y	Y	Y	Y	Y	Y	Y
3. Was the research design appropriate to address the aims of the research?	Y	Y	Y	Y	Y	Y	Y	Y	Y	Y
4. Was the recruitment strategy appropriate to the aims of the research?	Y	Y	U	Y	U	Y	Y	Y	Y	Y
5. Was the data collected in a way that addressed the research issue?	Y	Y	Y	Y	Y	Y	Y	Y	Y	Y
6. Has the relationship between researcher and participants been adequately considered?	N	N	N	N	N	N	N	N	Y	N
7. Have ethical issues been taken into consideration?	Y	U	Y	Y	N	Y	Y	U	Y	Y
8. Was the data analysis sufficiently rigorous?	Y	Y	Y	Y	Y	Y	Y	Y	Y	Y
9. Is there a clear statement of findings?	Y	Y	Y	Y	Y	Y	Y	Y	Y	Y
10. How valuable is the research?	Y	Y	Y	Y	Y	Y	Y	Y	Y	Y
11. Are the study's theoretical underpinnings (e.g., ontological and epistemological assumptions; guiding theoretical frameworks) clear, consistent and conceptually coherent?	N	Y	N	N	N	N	N	N	N	Y

Abbreviations: N, no; U, unstated or unclear; Y, yes.

### 2.5 Synthesis

A synthesis was performed to summarise the findings of the systematic review using a thematic approach due to the qualitative nature of the studies collated. This comprised three stages as outlined in Thomas and Harden (2008): coding text, developing descriptive themes and finally generating analytical themes. The primary researcher (GP) closely familiarised themselves with the content of the papers before generating preliminary codes, descriptive themes and analytical themes. This was then reviewed by two secondary team members (AT and RT) and the final themes and subthemes agreed upon.

Thematic synthesis is inherently interpretative, aiming to comprehend how a narrative is structured and the meanings it conveys, often within a particular cultural, social or personal context. Thematic synthesis allows for an explicit link between conclusions and the text of the included studies, providing transparency in the systematic reviewing process. This review was intended to provide a foundation to inform further research in this area, given the limited scope of existing research on offenders with ID. As this review is the first to explore and synthesise professional attitudes towards offenders with ID, a broad range of studies using a range of designs and outcome measures were included as well as a wide scope of concepts relating to professionals' attitudes and perceptions.

## 3 Results

### 3.1 Study Characteristics

Across the 10 papers identified, the total number of participants was 766, comprising police officers of varying ranks (*n* = 596); professionals working in voluntary organisations, specialist ID services or as appropriate adults (*n* = 71); mental health professionals working within the CJS (*n* = 41); probation officers (*n* = 12); solicitors/legal advisors (*n* = 12); magistrates (*n* = 11); judges (*n* = 9); barristers (*n* = 7); diversion panel members (*n* = 2); legal academics (*n* = 2); a prison worker (*n* = 1); and nonspecified CJS professionals (*n* = 5). Please note that the total is greater than the overall N because three participants held dual roles in one study (Chadwick and Wesson 2020). Studies were obtained from only four countries: the United Kingdom (*k* = 4); the United States (*k* = 3); Australia (*k* = 2); and the Republic of Ireland (*k* = 1) and publication years spanned from 2005 to 2023. Participants were recruited from a range of settings, including police teams, probation services, mental health provision within the CJS, social care services, law firms, judiciary, magistrate services and voluntary organisations. Of the 10 included papers, data collection methods included mixed methods survey (*k =* 3), secondary analysis of previously collected qualitative research data (*k =* 1), qualitative survey (*k =* 1), semistructured interviews (*k =* 3), semistructured focus groups (*k =* 1) and unstructured interviews (*k =* 1). Two studies utilised mixed populations including professions not originally identified for inclusion in the review (Chadwick and Wesson 2020; Gulati et al. 2021). It was not possible to extract data solely pertaining to nonhealthcare CJS staff; therefore, all data from the studies have been included in the synthesis. See Table 2 for a summary of study characteristics.

**TABLE 2:** Study characteristics.

Authors	Year	Title	Aims of Study	Country	Sample Size	Sample Characteristics	Setting	Data Collection Method	Analysis Method
Modell and Mak	2008	A Preliminary Assessment of Police Officers' Knowledge and Perceptions of Persons With Disabilities	To understand police officers' knowledge and perceptions of people with disabilities.	USA	124	Police officers (*n* = 124)	Police teams	Survey (mixed methods)	Content analysis
Chadwick and Wesson	2020	‘Blocked at Every Level’: Criminal Justice System Professionals' Experiences of Including People With Intellectual Disabilities Within a Targeted Magistrates' Court	To understand the experiences of professionals involved in the running of a targeted services Magistrates' court of including those with ID.	UK	46^a^	Probation (*n* = 12), Police officers (*n* = 6), Intellectual disability service professionals (*n* = 6), Specialist mental health providers working in courts and custody (*n* = 5), magistrates (*n* = 5), legal advisors/justices clerk (*n* = 4), defence lawyers (*n* = 3), mental health service professionals (*n* = 3), diversion panel member (*n* = 2), prosecution lawyer (*n* = 1), prison worker (*n* = 1), other CJS (*n* = 1)	Probation services, police teams, mental health, social care, court and tribunal services, voluntary organisations	Secondary analysis of qualitative data collected as part of an evaluative participatory action research investigation	Thematic network analysis
Gulati et al.	2021	Challenges for People With Intellectual Disabilities in Law Enforcement Interactions in Ireland; Thematic Analysis Informed by 1537 Person‐Years' Experience	To identify the unique challenges which PWID face in their interactions with LEOs in Ireland.	Ireland	95	People working in a voluntary or representative organisation for PWID (board members [ *n* = 4], organisation solicitor [*n* = 1], outreach service leader [*n* = 1], human resource manager [*n* = 1], project manager for mental health and ID [*n* = 1], administrator [*n* = 1], advocacy workers [*n* = 4], nurses [*n* = 3], speech and language therapist [*n* = 1], social workers [ *n* = 16], clinical psychologists [ *n* = 8], psychiatrist [*n* = 1]); people working in healthcare (psychiatrists [*n* = 12], a forensic psychiatrist [*n* = 1], nurses [ *n* = 7], clinical psychologists [*n* = 6], social worker [*n* = 2], healthcare chaplain [*n* = 1], healthcare solicitor [*n* = 1], speech and language therapist [*n* = 1]); and people working in law enforcement (Gardaí [*n* = 5], airport police/fire service officers [*n* = 2], judges [*n* = 2], barristers [*n* = 3], Solicitors [*n* = 8] and legal academics [*n* = 2])	Representative organisations, voluntary organisations, healthcare, police teams, lawyers, judiciary.	Survey (qualitative)	Thematic analysis
Diamond and Hogue	2022	Law Enforcement Officers: A Call for Training and Awareness of Disabilities	To explore the current perspectives of LEOs regarding PWID and to identify the current training needs of LEOs regarding disability awareness and interactions with PWID.	USA	13	Police officers (officer [*n* = 7], detective [*n* = 2], sergeant [*n* = 1], deputy [*n* = 1] and lieutenant [*n* = 2])	Police teams	Semistructured focus groups	Thematic analysis
Hellenbach	2011	Learning disabilities and criminal justice: custody sergeants' perceptions of alleged offenders with learning disabilities	To understand attitudes and opinions shared by custody sergeants regarding how ID might be conceptualised within the context of criminal justice.	UK	14	Custody sergeants (*n* = 14)	Police teams	Unstructured interviews	Grounded theory
Henshaw and Thomas	2011	Police encounters with people with intellectual disability: prevalence, characteristics and challenges	To understand the experiences and perceptions of operational members of the police in relation to their contacts with PWID.	Australia	226	Police officers (senior constable [*n* = 62], leading senior constable [ *n* = 62], constable [ *n* = 55], sergeant [ *n* = 34], senior sergeant [*n* = 8], protective services officer [*n* = 3] and inspector [*n* = 2])	Police teams	Survey (mixed methods)	Thematic analysis (for qualitative data)
Eadens et al.	2016	Police officer perspectives on intellectual disability	To examine police officer perceptions about PWID.	USA	188	Police officers ( *n* = 188)	Police teams	Survey (mixed methods)	Thematic analysis (for qualitative data)
Gendle and Woodhams	2005	Suspects who have a learning disability: Police perceptions towards the client group and their knowledge about learning disabilities	To understand the perceptions of police officers towards PWID and their knowledge of issues related to ID.	UK	8	Police sergeants (*n* = 8)	Police teams	Semistructured interviews	Content analysis
Cant and Standen	2007	What professionals think about offenders with learning disabilities in the criminal justice system	To explore the attitudes of professionals in the criminal justice system to PWID.	UK	28	Custody sergeants (*n* = 7), appropriate adults (*n* = 6), community psychiatric/forensic liaison nurses (*n* = 2), magistrates (*n* = 6) and Crown Court judges (*n* = 7)	Police teams, appropriate adult schemes, judiciary, magistrates services.	Semi‐structured interviews	Thematic analysis
Richards and Ellem	2018	Young people with cognitive disabilities and overrepresentation in the criminal justice system: service provider perspectives on policing	To explore the experiences of young people with ID's interactions with the police.	Australia	21	Professionals working in legal services (*n* = 4), professionals working in voluntary organisations and specialist services (*n* = 17)	Legal services, youth services, disability support services, specialist support services.	Semi‐structured interviews	Thematic analysis

Abbreviation: LEOs, law enforcement officers.

^a^ Three participants in this study held dual roles.

### 3.2 Synthesis

**FIGURE 3:**
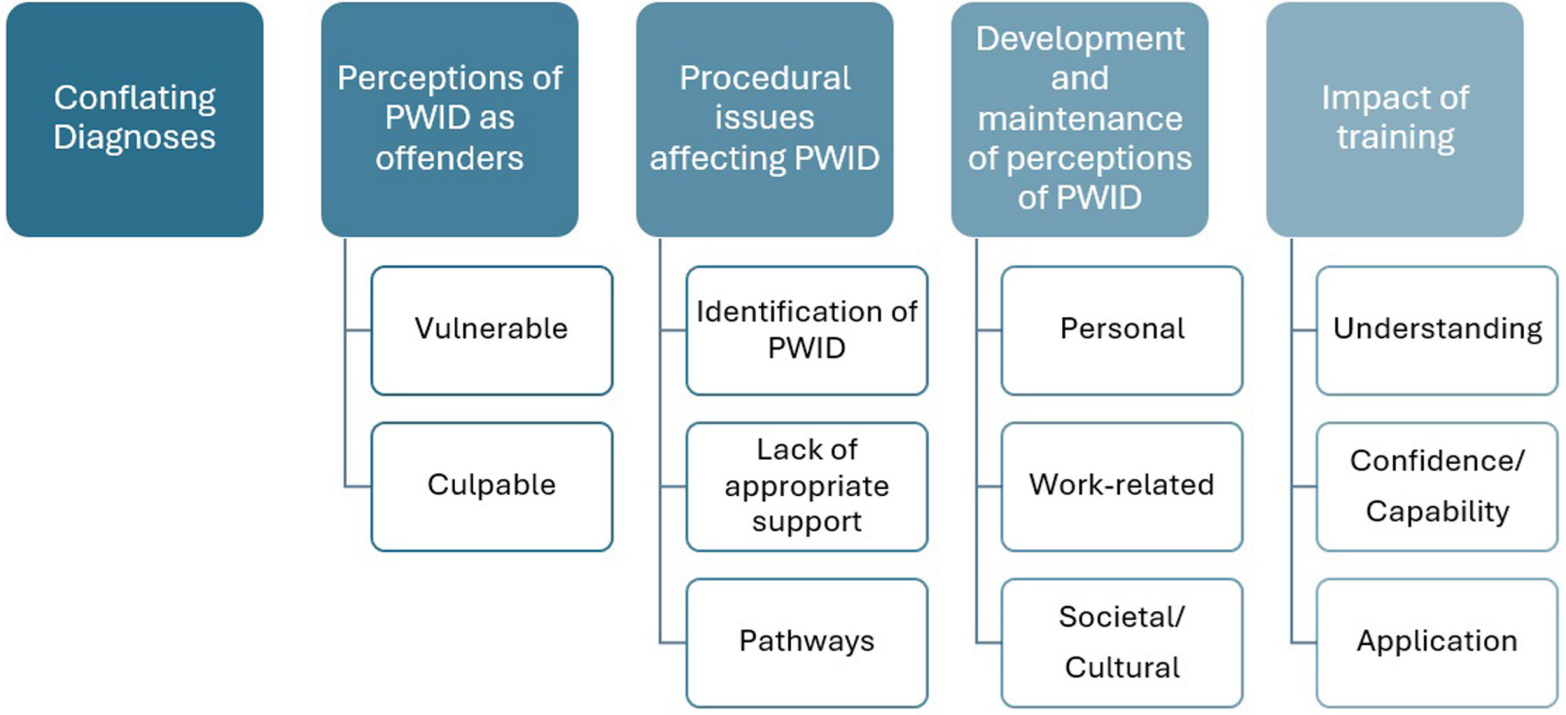
Themes and subthemes identified through data analysis.

Five major themes were identified through qualitative synthesis—*conflating diagnoses, perceptions of PWID as offenders, procedural issues affecting PWID, development and maintenance of perceptions of PWID* and *impact of training*—shown in Figure 3. Themes and subthemes are presented with illustrative quotes in Table 3.

**TABLE 3:** Thematic synthesis*.*

Theme	Subthemes	Quotations
Conflating diagnoses		‘Are emotional disabilities where the freaks cry a lot?’ (Modell and Mak 2008, 186)
		‘I think a lot of clients […] do not wish to be labelled’. (Chadwick and Wesson 2020, 138)
		‘Anxiety which can lead to changed/heightened/inappropriate behaviour, that can be misinterpreted by the officer’. (Gulati et al. 2021, 5)
		‘My understanding of it would be, it would have to necessarily be something that actually impairs somebody's ability to understand or to communicate while they are here. So for instance you have the schizophrenic who is very well controlled on medication and who presents normally politeness’. (Hellenbach 2011, 17)
		‘He cannot read, so I definitely think he had learning disabilities’. (Gendle and Woodhams 2005, 76)
Perceptions of PWID as offenders	Vulnerable	‘Responses indicated that police officers see persons with disabilities as victims of crimes more often than as perpetrators’. (Modell and Mak 2008, 186)
		‘We felt it was very important (for offenders with ID) to be treated equally under the law […] they either get the chat in the back of the police car. This is literally a little chat saying, this is very bad, you were very naughty and do not do it again, they resisted taking people to the police station. They get a caution. Or it gets diverted’. (Chadwick and Wesson 2020, 137)
		‘We're not suggesting that people who have got a learning disability get preferential treatment but we are acknowledging the fact that their condition […] may (be) one of the leading factors for why they have offended, repeat offending or why they cannot get out of that cycle’. (Chadwick and Wesson 2020, 140)
		‘People with ID are very vulnerable to suggestion and coercion without adequate advocacy’. (Gulati et al. 2021, 5)
		‘Being more empathetic and more personable and stepping back from that harder line that we typically take’. (Diamond and Hogue 2022, 229)
		‘I suppose in some ways they [people who have learning disabilities] could be more easily influenced than someone with a bit more common sense’. (Gendle and Woodhams 2005, 75)
		‘If you are talking about someone with some major learning difficulties, for instance someone with Down's syndrome, who you could blatantly tell was extremely upset and they had been and nicked a packet of Smarties because they did not have any money and they were hungry, you would be taking them home, you know’. (Gendle and Woodhams 2005, 76)
		‘See whether they understand and are as capable as anybody else. If you can normalise them there then you have to treat them the same, but if you cannot then I think there's got to be some sort of positive discrimination’. (Cant and Standen 2007, 177)
		‘The foetal alcohol syndrome and things like that … diminish his capacity to understand, when he's in an emotional state, the consequences of his actions’. (Richards and Ellem 2018, 162)

	Culpable	‘If they have been assessed, if they have committed a crime and they have been assessed if they have been criminally responsible then of course they should be punished. They should go to prison like anyone else. You cannot commit a crime and get away with it because you have a learning disability. As long as they know the difference between right and wrong and they understood the process, and the evidence has been secured and preserved’. (Hellenbach 2011, 19)
		‘If he has done it and just because he is not bright enough to have an intelligent argument about whether he is guilty or not. Does it matter? If he is guilty and he has done it and he could not defend himself cause he wasn't bright enough to. OK you did it, go to prison. While the next person might be able to argue their way out of it because they were brighter. I have not got much sympathy really. As opposed to people who are very bright and dream up all sorts of excuses, defences that are fictional and then get away with things’. (Hellenbach 2011, 19)
		‘You've got to make sure you are not giving too many advantages, or unjustified advantages to defendants. You must not make it less easy for the Crown to get a conviction’. (Cant and Standen 2007, 177)
‘Sometimes the police can be a bit skeptical of … what they perceive to be excuses’. (Richards and Ellem 2018, 164)	
Procedural issues affecting PWID	Identification of PWID	‘Appropriate identification of cases those with MH issues are a lot easier for the police to identify and flag up than those with learning disability’. (Chadwick and Wesson 2020, 138)
		‘I've a concern that there are people we do not recognize’. (Gendle and Woodhams 2005, 76)
		‘The question is, would it strike one sitting in court that somebody has a learning disability as an obvious thing and the answer is that no it would not’. (Cant and Standen 2007, 176)
		‘Generally speaking the police do not identify those young people as having an impairment’. (Richards and Ellem 2018, 164)
		‘The difficulty is that someone has to have (that) flagged up, so if nobody tells you then you would not treat them differently’. (Chadwick and Wesson 2020, 140)
	Lack of appropriate support	‘The systems are not set up to be accessible for people (with ID), people are blocked at every level, right from the point where the police get involved through diversion, the court process, they are so complex and operated by people who do not have many dealings with people with ID’. (Chadwick and Wesson 2020, 140)
		‘There should be a stronger link between those on the frontline of law enforcement and disability services’. (Gulati et al. 2021, 5)
		‘Gardaí need training but also advocates for person with intellectual disability when person becomes involved in the system’. (Gulati et al. 2021, 5)
		‘You try to listen and ask family members around what exactly is wrong with him. How can we approach to get through to him?’ (Diamond and Hogue 2022, 230)
		‘Behaviour is unpredictable—difficult to plan a response’. (Henshaw and Thomas 2011, 626)

	Pathways	‘(Working with people with ID) it's not planned, there's no pathway that says this is what you do the first time you come into contact’. (Chadwick and Wesson 2020, 137)
		‘I would not ask the doctor to see somebody who was just not bright. I would just say, is there somebody who could come over with you when you are interviewed?’ (Hellenbach 2011, 17)
		‘It is all pressures on you which can make you cut corners or lead you to cut corners if you wanted to and making mistakes’. (Hellenbach 2011, 20)
		‘It's in the codes of practice, this book with policies and procedures in, anyone you suspect has got learning disabilities, you get an appropriate adult’. (Gendle and Woodhams 2005, 75)
		‘Sometimes you make the wrong decision. Sometimes you have [an appropriate adult] in there when you do not need them and sometimes you do not have them in when you need them’. (Gendle and Woodhams 2005, 78)
[re: Challenges faced in resolving encounters with people with intellectual disability] ‘Identifying what services I can use to help me’. (Henshaw and Thomas 2011, 626)	
Development and maintenance of perceptions of PWID	Personal	‘Very little training, very little help from anybody along the way. We just kind of learned as we went, as a parent’. (Diamond and Hogue 2022, 229)
	Work related	‘[…] somebody committed an offence (and) has not been charged and interviewed. Because the Police are hung up on the fact that she might not have the capacity without knowing what kind of capacity they are worried about […] They took the witness statement, so there's a crime number for the victim and then they just sent him back home’. (Chadwick and Wesson 2020, 139)
		‘I would not have said really bad learning difficulties, I've never seen anyone like that’. (Chadwick and Wesson 2020, 139)
		‘It's all on‐the‐job training really’. (Gendle and Woodhams 2005, 79)
		‘I try to get them [the officers in training] to stop in for a cup of tea and speak to the staff and get used to being around the people with learning disabilities and we certainly know people with learning disabilities in our area. For quite a while now it's been flavour of the month (referring to multiagency working)’. (Gendle and Woodhams 2005, 79)
		‘You can tell by the look on their face that they have not got a clue (referring to a person with learning disabilities not understanding their rights)’. (Gendle and Woodhams 2005, 76)
		‘If he does not understand something, he's more aggressively reactive, and does not really kind of explain that he does not understand what's going on, he just starts shouting … because he does react so strongly … his interactions with police when he is doing that is they get harder on him’. (Richards and Ellem 2018, 163)
		‘People with an intellectual disability … and I know it sounds awful but they lie a lot … and that is because they have had to. “Yes, I can read that.” “Yes, I am okay.” “Yes I … did not do that to Johnny.” “Yes I'll do that” … they just do not want people to notice that they are missing, you know, some sort of … some sort of information that's been imparted. So they'll say yes to everything’. (Richards and Ellem 2018, 164)
		‘[Police] take things at face value … they take what they hear but they do not look at the cognitive [capacity] of the person’. (Richards and Ellem 2018, 165)
		‘A culture of respect and understanding of the complexities involved needs to be fostered at management level’. (Gulati et al. 2021, 5)
	Societal/cultural	‘For Question 5 (“What does the term autism mean to you?”) … more than 35% listed simply “Rain Man” as their response’. (Modell and Mak 2008, 186)
Impact of training	Understanding	‘A police officer should have the skills and knowledge of disabilities so that officer does not mistake a non‐compliant person for a person with a disability’. (Modell and Mak 2008, 186)
		‘It's not going to work for every single scenario, but 9 times out of 10 if you can try to bring yourself to that level of that person, try to empathize, try to determine what's going on with that person, try to figure out what the crisis might be, or whatever the situation is, just trying to be empathetic. I've seen, at least in my experience, that goes a long way’. (Diamond and Hogue 2022, 229)
	Confidence/capability	‘I felt a lot more confident (after training) dealing with those types of cases plus it meant that you were able to advise the magistrates much more easily than we were before as well’. (Chadwick and Wesson 2020, 139)
		“Our officers are ill‐quipped, ill‐trained to handle some of these [situations with PWD].” (Diamond and Hogue 2022, 231)
	Application	‘If people aren't coming through, people aren't getting a chance to use their training, that sort of training will eventually evaporate’. (Chadwick and Wesson 2020, 189)
		‘Placement opportunities during training in services for disabilities may increase understanding and learning for law enforcement officers’. (Gulati et al. 2021, 5)
		‘You get a little scratch on the surface. Here and there are some disabilities you'll be called [about]. Called to a scene where there's some disability involved [but] I think it's more along the lines of mental health. And you have got crisis intervention training (CIT) as well’. (Diamond and Hogue 2022, 231)
		‘In the training we need to be shown examples. All of our officers have seen autistic people, people with Down syndrome … So, I think we might need to be shown visually, rather than told. We might need to be shown some typical interactions, whether they are staged or real’. (Diamond and Hogue 2022, 231)

#### 3.2.1 Conflating Diagnoses

CJS staff cannot reliably differentiate between mental health difficulties, autism and ID (Chadwick and Wesson 2020; Gendle and Woodhams 2005; Gulati et al. 2021; Hellenbach 2011; Henshaw and Thomas 2011; Modell and Mak 2008; Richards and Ellem 2018). There appeared to be a pattern of reported prioritisation of mental health conditions over ID at all levels of contact with the CJS from training to identification and ongoing support. The overshadowing of ID by mental health conditions could be partially attributed to legislation in which issues pertaining to mental health and ID are encompassed under the umbrella of ‘mental disorders’, such as the Mental Health Act (MHA; 1983) and the Police and Criminal Evidence Act (PACE; 1984) in the United Kingdom, the Criminal Code Act (1995) in Australia and Title II of the Americans with Disabilities Act in the United States. This can also be reflective of the differing definitions of ID across systems and countries. However, it could also be reflective of the potential invisibility of ID compared to aspects of certain mental health conditions. For instance, one might observe someone who is experiencing psychosis to be interacting with hallucinations or someone acutely depressed to be self‐harming. It could be the case that offenders who are quiet and compliant in their cells or hospital beds are less likely to be noticed, as their symptoms do not cause more immediate issues for the institution resulting in less priority given to training CJS professionals around these issues.

#### 3.2.2 Perceptions of PWID as Offenders

Perceptions of CJS staff were divided, with offenders with ID perceived as either equally or less culpable than their non‐ID counterparts. This resulted in two subthemes: *vulnerable* and *culpable*.

##### 3.2.2.1 Vulnerable

Those who perceived PWID as less culpable instead highlighted their vulnerability (Cant and Standen 2007; Gendle and Woodhams 2005; Gulati et al. 2021; Modell and Mak 2008; Richards and Ellem 2018) and mentioned diversion of such offenders from the CJS and into specialist services (Cant and Standen 2007; Chadwick and Wesson 2020; Gendle and Woodhams 2005). However, this poses significant issues given the lack of reliability in identifying and supporting offenders with ID. Some participants spoke of treating offenders with ID ‘equally’ under the law while simultaneously evidencing diversionary tactics and leniency applied when dealing with offenders with ID, suggesting that treatment is deemed ‘equal’ when in fact it is ‘equitable’.

##### 3.2.2.2 Culpable

Those who perceived PWID as equally culpable as non‐ID offenders frequently referred to justice as the driving factor for prosecuting offenders with ID (Cant and Standen 2007; Hellenbach 2011). There was a sense that part of one's professional identity was to be victim‐focused and justice‐driven by enforcing the law as it is written and not affording undue leniency to offenders with ID (Cant and Standen 2007; Richards and Ellem 2018). Some participants cited systemic demands such as ‘ensuring ease of conviction’ (Cant and Standen 2007, 177), whereas others described ID as being perceived as ‘excuses’ (Richards and Ellem 2018, 164) or ‘illegitimate mitigation of … wrongdoing’ (Hellenbach 2011, 18). This is potentially concordant with the lack of understanding of what ID actually entails and how it can present in individuals who may not fit society's stereotyped expectations of a PWID.

#### 3.2.3 Procedural Issues Affecting PWID

There continues to be organisational barriers that prevent PWID from accessing appropriate support throughout the CJS, encompassing first contact with CJS and identification of PWID to onward pathways and support through the courts.

##### 3.2.3.1 Identification of PWID

There appeared to be a sense of recognition that PWID are underidentified and undersupported throughout the CJS (Cant and Standen 2007; Chadwick and Wesson 2020; Gendle and Woodhams 2005; Hellenbach 2011; Richards and Ellem 2018), and this was complemented by a distinct lack of belief in the efficacy of existing systems (Chadwick and Wesson 2020; Richards and Ellem 2018). There was a sense of reliance on an individual professional's ability to identify a PWID based on stereotypical indicators, such as appearance, communication and social background (for instance, offenders whose residence was a supported living facility) rather than using any formal, predetermined screening criteria (Eadens et al. 2016; Henshaw and Thomas 2011; Richards and Ellem 2018). Participants also spoke of reliance on PWID having been ‘flagged’ by other systems or organisations before coming into contact with certain CJS services (Chadwick and Wesson 2020) or after coming into contact with frontline CJS professionals and progressing through the system (Cant and Standen 2007; Hellenbach 2011). This poses a significant concern, as this creates gaps in support due to a lack of identified responsibility and insufficient protocol for identifying PWID.

##### 3.2.3.2 Lack of Appropriate Support

This subtheme captures the inadequacies of support within the CJS, encompassing both formal and informal sources of support. Existing approaches appear to rely on family members or familiar adults when PWID come into contact with the CJS (Diamond and Hogue 2022; Gendle and Woodhams 2005). Participants reflected on the need for specific training (Chadwick and Wesson 2020; Gulati et al. 2021; Henshaw and Thomas 2011), advocates embedded within the CJS (Gulati et al. 2021) and stronger links between the CJS and specialist disability services in order to better meet the support needs of PWID (Cant and Standen 2007; Chadwick and Wesson 2020; Gulati et al. 2021; Richards and Ellem 2018). There was a sense that the lack of appropriate support exacerbates stress for both PWID and CJS professionals.

##### 3.2.3.3 Pathways

It became apparent that there is a commonality of disorganisation of pathways for PWID in the CJS (Cant and Standen 2007; Chadwick and Wesson 2020; Gendle and Woodhams 2005; Gulati et al. 2021; Hellenbach 2011; Henshaw and Thomas 2011). Linked with the previous subtheme—lack of appropriate support—this subtheme is reflective of inadequate or nonexistent protocols within the CJS for working with PWID, meaning that individuals coming into contact with the CJS will have their experience dictated by the knowledge and skills of the professional in front of them as opposed to effective standardised approaches designed specifically to support PWID.

#### 3.2.4 Development and Maintenance of Perceptions of PWID

Understanding how perceptions of PWID are developed and maintained can support the identification of targeted changes to training and processes within the CJS. A lack of knowledge, experience and confidence in both working with PWID and the relevant processes within the CJS perpetuates assumptions that PWID lack capacity or understanding. The pervasive stigma attached to an ID label has appeared to create a culture of reluctance to access support for offenders for fear of causing insult, without adequate assessment of the necessity of such support.

##### 3.2.4.1 Personal

Personal experiences were idiosyncratic but highlighted as important and defining interactions, which supported professionals' understanding and attitudes towards PWID. This included children and adults with ID with varying degrees of familiarity with the participants, such as close family relations and children of colleagues or acquaintances (Diamond and Hogue 2022; Eadens et al. 2016).

##### 3.2.4.2 Work Related

Understandably, work‐related experiences of PWID were the most salient for participants and most frequently reported (Chadwick and Wesson 2020; Diamond and Hogue 2022; Eadens et al. 2016; Gendle and Woodhams 2005; Gulati et al. 2021; Hellenbach 2011; Henshaw and Thomas 2011; Richards and Ellem 2018). As experiences are repeated and patterns emerge throughout a professional's career, whether positive or negative, perceptions of PWID are developed and maintained. If there is an organisational culture of highly pressured, rushed processes then due care may not be taken on an individual level to challenge one's existing biases (Hellenbach 2011). Alternatively, where systems are organised to tread carefully and assumptions are not made without adequate investigation or evidence, PWID are likely to be identified, supported and, most crucially, respected during their contact with the CJS (Gulati et al. 2021).

##### 3.2.4.3 Societal/Cultural

If a CJS professional has a limited scope of experience with PWID, they may become complacent when faced with individuals who do not ‘fit’ the professional's preconceived notions of who is or is not a PWID and what that means in terms of a person's abilities and support needs. This results in milder or highly masked PWID slipping under the radar, remaining unidentified in the CJS, and therefore not being afforded the support they require and deserve. This subtheme captures how a high degree of participants from one study related their entire understanding of a diagnosis to a popular film, which has a very nuanced depiction of one specific type of developmental disability (Modell and Mak 2008).

#### 3.2.5 Impact of Training

This theme encompasses the impact of prior training on existing understanding of PWID and identifies a continuing need to upskill and educate CJS staff. Training needs identified throughout the studies encompassed identification of symptoms, basic knowledge of characteristics of disability, access to resources and communication skills (Chadwick and Wesson 2020; Gulati et al. 2021; Modell and Mak 2008). The inadequacy of current training was supported by descriptions of it as vague, basic and minimal (Eadens et al. 2016; Modell and Mak 2008) as well as voluntary, superficial and not memorable (Diamond and Hogue 2022) resulting in CJS staff perceiving themselves as competent when they may not have been (Henshaw and Thomas 2011; Modell and Mak 2008).

##### 3.2.5.1 Understanding

A key shift identified by professionals following training was in their understanding of PWID. Participants related their improved understanding to being able to identify a PWID more easily and how they might adjust their approach in situations involving PWID (Diamond and Hogue 2022; Gulati et al. 2021; Modell and Mak 2008).

##### 3.2.5.2 Confidence/Capability

Another facet of this theme was professionals' confidence and capability in working with PWID. Professionals who had undergone impactful training on working with PWID reported greater confidence in their ability to work effectively with PWID in the CJS (Chadwick and Wesson 2020). Conversely, professionals who had only undergone the basic training required of their role, service or organisation reflected on feeling ill‐equipped to appropriately manage cases related to PWID (Diamond and Hogue 2022).

##### 3.2.5.3 Application

Although training was reported to positively impact upon attitudes towards PWID, the findings suggest that professionals' feel that their skills and confidence in working with PWID will diminish without opportunities to put their training into practice (Chadwick and Wesson 2020). It was suggested that placement opportunities (Gulati et al. 2021) and experiential training (Diamond and Hogue 2022; Gendle and Woodhams 2005; Gulati et al. 2021) could help professionals to retain and refine their skills and knowledge.

## 4 Discussion

This qualitative systematic review offers an exploration into CJS professionals' perceptions of PWID, specifically those who come into contact with the CJS as offenders. Unlike previous reviews, this systematic review highlights the views of professionals throughout all stages of the CJS and explores how attitudes are developed and maintained by professionals' experiences of PWID, societal expectations of PWID and systemic and organisational barriers to the effective involvement of PWID in the CJS. By using thematic synthesis, the present review identified that CJS professionals' views and attitudes towards PWID were influenced by personal and work‐related experiences, societal stereotypes and attributions, and training. The views of CJS professionals towards PWID are not homogenous and were in fact highly divided at all levels from basic understanding of PWID to attitudes towards justice in cases where the alleged offender is a PWID. A cumulative effect can be observed in the emerged themes of this review; the lack of adequate training, reliance on informal and potentially inaccurate knowledge of ID, the limited support and pathways available within the CJS, and the divisive perceptions of PWID as offenders all significantly impact on professionals' ability to effectively understand and support PWID who come into contact with the CJS and serves to perpetuate existing, often negatively biassed, views of PWID.

As research suggests that police officers perceive people with mental disorders as more dangerous than the general population (Lamb et al. 2002), this could result in greater use of force by frontline law enforcement when dealing with PWID. People with disabilities, whether developmental, intellectual or psychiatric, already account for approximately one third of deaths in fatal interactions with law enforcement (Perry and Carter‐Long 2016). This exemplifies the stark disadvantages faced by PWID in their contact with the CJS. Hayes et al. (2007) point out that identification of offenders with ‘borderline’ ID in particular is compromised by the absence of institutional systems that flag up an individual's support needs as the majority of such individuals have had no previous contact with specialist services. Therefore, the lack of clear processes and pathways both fails to identify and fails to support offenders with ID throughout the CJS. A reliance on offenders with additional needs having already been flagged earlier in their contact with the CJS creates complacency which allows PWID to slip through the organisational cracks undetected and unsupported. Identifying offenders with ID or other additional needs is crucial for ensuring that their rights are recognised and meaningfully met throughout their contact with the CJS (Gulati, Cusack, et al. 2020; Gulati, Kelly, et al. 2020).

Training for CJS professionals aids in challenging misperceptions, stereotypes and negative biases (Bailey et al. 2001; Gardner et al. 2018; Henshaw and Thomas 2011). However, the findings of this review suggest that training needs to be specific to working with PWID and incorporate experiential elements to be most impactful and improve retention (Chadwick and Wesson 2020; Diamond and Hogue 2022; Gulati et al. 2021). Training led to changes in professionals' understanding and approaches when working with PWID (Chadwick and Wesson 2020), which serves to better uphold the human rights of PWID as they progress through the CJS. However, the findings also show that professionals' skills and confidence in working with PWID will diminish if not afforded opportunities to put their training into practice (Chadwick and Wesson 2020). It is therefore paramount that CJS professionals have regular training refresher courses to keep their skills sharp and their knowledge up to date so that they are able to best serve PWID who come into contact with their services.

### 4.1 Strengths and Limitations of the Current Review

The overall quality of the included studies was satisfactory, though there was a general lack of reflexivity regarding the relationship between the researchers and participants and very few studies with clearly outlined theoretical underpinnings. Given the subjective nature of qualitative research, these are key factors to consider as they speak to the ways in which the researchers construct knowledge and understand their findings. The data collection methods used throughout the included studies were varied but of sufficient quality and rigour, with several researchers employing pilot studies to ensure the validity of survey and interview questions ahead of data collection.

The qualitative synthesis utilised for this review could be considered a limitation as themes have been developed without the original context of the coded quotes. Inclusion criteria limited this review to studies written in English and based in countries with a Common Law system. This therefore limits the review to a representation of Western, Anglicised CJS which limits the generalisability of this review. Searches were conducted on primarily health and medical related databases, and the majority of initial screening was conducted by only one reviewer. This potentially acts as a limitation as some evidence may not have been captured, such as from criminal justice databases, and the opportunity for oversight from additional reviewers was reduced.

## 5 Conclusions and Recommendations

PWID may experience discrimination as CJS professionals may not regard them as credible and therefore may not fully investigate crimes committed against them or by them due to pervasive stereotypes and misattributions about PWID. CJS professionals who had received specific training felt that they were more prepared for interactions with PWID (Gardner et al. 2018; Henshaw and Thomas 2011); however, the inadequacy of current training was supported by the descriptions of it as vague, basic and minimal. There was also variability in the reported adaptations and adjustments made for PWID in the CJS. Training for CJS professionals should be specific to ID and incorporate an experiential element for greater impact and retention (Diamond and Hogue 2022; Gulati et al. 2021), and efforts should be made at organisational levels to create clear processes and pathways to reduce confusion and formalise the procedures for identifying and supporting PWID in the CJS.

It would be of benefit to gain a greater understanding of how professional identity interplays with perceptions of PWID; the current review was not able to meaningfully distinguish between the views of frontline CJS professionals, such as police officers, and professionals in the later stages of a case's progression through the CJS, such as judges and magistrates, as the majority of the sample was police based. Given the onus lies with frontline staff for identification of PWID and commencement of appropriate protocols (where such procedures exist), it could be that professionals working in the later stages of the CJS are less exposed to and less knowledgeable about PWID. Future reviews should also seek to understand the views and attitudes of CJS professionals across a greater diversity of countries and CJS, particularly developing nations, as this would identify cultural differences that influence professionals' perceptions of PWID.

## Data Availability

The data of this study can be available from the authors upon request.

## Appendix

https://onlinelibrary.wiley.com/page/journal/13652788/homepage/forauthors.html

### Systematic Reviews

The maximum word length for systematic reviews is 6000 words. Authors submitting a systematic review are encouraged to assess the quality of their reporting against the PRISMA checklist prior to submission (http://www.prisma‐statement.org/2.1.2—PRISMA 2009 Checklist.pdf) or MOOSE guideline (https://www.equator‐network.org/reporting‐guidelines/meta‐analysis‐of‐observational‐studies‐in‐epidemiology‐a‐proposal‐for‐reporting‐meta‐analysis‐of‐observational‐studies‐in‐epidemiology‐moose‐group/). Submitted systematic reviews are expected to adhere to these guidelines, and checklists should be submitted alongside manuscripts as Supporting Information for review. Manuscripts may be returned to authors if checklists are missing from the submission.

### Preparation of the Manuscript

#### Author Services

Prior to submission, we encourage you to browse the ‘Author Resources’ section of the Wiley ‘Author Services’ website (https://authorservices.wiley.com/author‐resources/Journal‐Authors/index.html). This site includes useful information covering such topics as copyright matters, ethics and electronic artwork guidelines.

#### Free Format Submission

The Journal of Intellectual Disability Research now offers free format submission for a simplified and streamlined submission process.

Before you submit, you will need:

- Your manuscript: This can be a single file including text, figures and tables or separate files—whichever you prefer. All required sections should be contained in your manuscript, including abstract, introduction, methods, results and conclusions. Figures and tables should have legends. References may be submitted in any style or format, as long as it is consistent throughout the manuscript. If the manuscript, figures or tables are difficult for you to read, they will also be difficult for the editors and reviewers. If your manuscript is difficult to read, the editorial office may send it back to you for revision.
- The title page of the manuscript, including statements relating to our ethics and integrity policies (if applicable):
  ○ funding statement
  ○ conflict of interest disclosure
  ○ data availability statement
  ○ ethics approval statement (for studies involving animal subjects and/or human participants)
  ○ permission to reproduce material from other sources

#### Writing for Search Engine Optimization

Optimise the search engine results for your paper, so people can find, read and ultimately cite your work. Simply read our best practice SEO tips—including information on making your title and abstract SEO‐friendly and choosing appropriate keywords.

#### Presubmission English‐language Editing

Authors for whom English is a second language may choose to have their manuscript professionally edited before submission to improve the English. Visit our site to learn about the options. All services are paid for and arranged by the author. Please note using the Wiley English Language Editing Service does not guarantee that your paper will be accepted by this journal.

#### Spelling

- Spelling should conform to the Concise Oxford Dictionary of Current English.
- A high proportion of papers are submitted with the term ‘behavior’ as opposed to ‘behaviour’; please use ‘behaviour’.
- Where applicable, the journal standard is to use words ending in ‐ise as opposed to ‐ize. For example, use ‘analyse’ and ‘standardise’ as opposed to ‘analyze’ and ‘standardize’.

Units of measurements, symbols and abbreviations should conform with those in Units, Symbols and Abbreviations (1977) published and supplied by the Royal Society of Medicine. This specifies the use of SI units.

#### Terminology

It is important that the term ‘intellectual disabilities’ or ‘intellectual disability’ is used when preparing manuscripts. The term ‘person’, ‘people’, ‘children’, ‘participant(s)’ or other appropriate term should be used as opposed to, for example, ‘patient(s)’.

#### *p* Values

Provide exact *p* values. *p* values should include up to two or three decimals (e.g., 0.05 or 0.002). *p* values smaller than 0.001 should be written as *p* < 0.001. Ensure that no *p* values are reported as *p* = 0.000 (should be *p* ≤ 0.001).

#### Optimising Your Paper on Social Media

If your paper is accepted for publication, we would like to present three, headline style summary statements on our Facebook and X feed. When you submit your article, you will be asked to enter up to three short headlines (key statements) capture the importance of your paper.

### Manuscript Structure

The manuscript should be submitted in separate files: title page; main text file; and figures.

A ‘title page’ must be submitted as part of the submission process as a ‘Supplementary File Not for Review’. The title page should contain

- a short informative title that contains the major key words. The title should not contain abbreviations (see Wiley's best practice SEO tips) and should normally be no longer than 15 words in length;
- the full names of the authors;
- the author's institutional affiliations at which the work was carried out;
- the full postal and email address, plus telephone number, of the author to whom correspondence about the manuscript should be sent;*.
- acknowledgements;
- conflict of interest statement.

The present address of any author, if different from that where the work was carried out, should be supplied in a footnote.

*On initial submission, the submitting author will be prompted to provide the email address and country for all contributing authors.

#### Acknowledgements

Contributions from anyone who does not meet the criteria for authorship should be listed (including any advisors/consultees with intellectual disability), with permission from the contributor, in an Acknowledgments section. See section on Authorship for more detail. Material support should also be mentioned Thanks to anonymous reviewers are not appropriate.

#### Conflicts of Interest

The authors declare no conflicts of interest.

#### Main text

As papers are double‐blind peer reviewed the main text file should not include any information that might identify the authors.

The main text of the manuscript should be presented in the following order: (i) structured abstract and key words (ii) text, (iii) references, (vi) endnotes, (vii) tables (each table complete with title and footnotes) and (ix) figure legends. Figures should be supplied as separate files. Footnotes to the text are not allowed and any such material should be incorporated as endnotes.

#### Abstract

For all submissions, a structured summary should be included at the beginning of the article, incorporating the following headings: background, method, results and conclusions. These should outline the questions investigated, the design, essential findings and the main conclusions of the study.

#### Keywords

The author should also provide up to six keywords. Please think carefully about the keywords you choose as this will impact on the discoverability of your paper during literature searches (https://authorservices.wiley.com/bauthor/seo.asp)

#### References

The journal follows the Harvard reference style.

References in text with more than two authors should be abbreviated to Brown et al. (1977).

Where more than six authors are listed for a reference, please use the first six and then ‘et al’.

Authors are encouraged to include the DOI (digital object identifier) for any references to material published online. See www.doi.org/ for more information. If an author cites anything which does not have a DOI they run the risk of the cited material not being traceable.

Authors are responsible for the accuracy of their references.

The reference list should be in alphabetical order thus:

Giblett E.R. 1969 Genetic Markers in Human Blood Blackwell Scientific Publications Oxford

Moss T.J. Austin G.E. 1980 Preatherosclerotic Lesions in Down's Syndrome Journal of Mental Deficiency Research 24 137 141

Seltzer M. M. Krauss M.W. 1994 Aging Parents With co‐Resident Adult Children: The Impact of Lifelong Caregiving Seltzer M. M. Krauss M.W. Janicki M. P. Life Course Perspectives on Adulthood and Old Age 3 18 American Association on Mental Retardation Washington, DC

#### Endnotes

Endnotes should be placed as a list at the end of the paper only, not at the foot of each page. They should be numbered in the list and referred to in the text with consecutive, superscript Arabic numerals. Keep endnotes brief; they should contain only short comments tangential to the main argument of the paper.

Tables should include only essential data. Each table must be typewritten on a separate sheet and should be numbered consecutively with Arabic numerals, for example, Table 1, Table 2, etc., and give a short caption.

Figure legends should be concise but comprehensive—the figure and its legend must be understandable without reference to the text. Include definitions of any symbols used and define/explain all abbreviations and units of measurement.

All illustrations (line drawings and photographs) are classified as figures. Figures should be numbered using Arabic numerals and cited in consecutive order in the text. Each figure should be supplied as a separate file, with the figure number incorporated in the file name.

#### Preparing Figures

Although we encourage authors to send us the highest quality figures possible, for peer‐review purposes we are happy to accept a wide variety of formats, sizes and resolutions. Click here for the basic figure requirements for figures submitted with manuscripts for initial peer review, as well as the more detailed post‐acceptance figure requirements.

#### Colour figures

Figures submitted in colour may be published in colour free of charge. Please note, however, that it is preferable that line figures (e.g., graphs and charts) are supplied in black and white so they are legible if printed by a reader in black and white.

The Supporting Information is information that is not essential to the article but that provides greater depth and background. It is hosted online and appears without editing or typesetting. It may include tables, figures, videos and datasets. Click here for Wiley's FAQs on supporting information.

Please note that the provision of supporting information is not encouraged as a general rule. However, the Supporting Information will be assessed by reviewers and editors and will be accepted if it is essential.
